# 6.1 STUDY OF ALTERED NEUROIMMUNITY IN PSYCHOSIS USING PET-BASED IMAGING OF THE TRANSLOCATOR PROTEIN 18 KDA: PROMISES, PITFALLS, AND FUTURE DIRECTIONS

**DOI:** 10.1093/schbul/sby014.018

**Published:** 2018-04-01

**Authors:** Jennifer Coughlin, Tina Notter, Yong Du, Martin Pomper, Akira Sawa, Urs Meyer

**Affiliations:** 1 Johns Hopkins University; 2 University of Zurich; 3 Johns Hopkins University School of Medicine

## Abstract

**Background:**

Successful development of high affinity radioligands for the translocator protein 18 KDa (TSPO) has contributed to a rapid rise in their use with positron emission tomography (PET) imaging to quantitatively detect the higher density of TSPO in neuropsychiatric conditions with putative microglial activation or reactive gliosis in vivo. [11C]PK11195 has been widely used to study TSPO in many neurological and psychiatric diseases, but the quality of quantified binding estimates using this first-generation radioligand is hampered by low signal to noise ratio, which also limits the sensitivity to detect group differences in binding. Second-generation radiotracers for TSPO such as [11C]DPA-713, [11C]PBR28, or [18F]FEPPA have superior specificity for the target and improved brain penetrance. However, in spite of these promising newer generation radioligands, their use with PET neuroimaging to study the immune response in psychotic diseases like schizophrenia has yielded inconsistent results of low, unchanged, or even tendency toward decreased binding to TSPO compared to data from controls.

**Methods:**

In this presentation, we will provide necessary biological and methodological perspective to help interpret better the recent results from imaging TSPO in psychosis. We will first review its expression by many cell types including activated microglia, the resident immune cells in the brain, and its diverse functional roles including TSPO as a biomarker of classic neuroinflammatory processes. We will then present optimized methods for estimating useful binding outcomes that go beyond correction for TSPO genotype to minimize effects of factors, including those related to the diverse roles of TSPO, which otherwise introduce limiting, inter-individual variability in binding.

**Results:**

These methods include the reporting of relative binding in one tissue to another, where such global factors are cancelled out by their appearance in the numerator and denominator of the outcome ratio. Use of this ratio approach may decrease inter-individual variability in binding measures and improve the sensitivity and statistical power to detect differences between cohorts. In contrast, use of a relative outcome measure may limit the utility of TSPO imaging since a difference between cohorts or within a subject over time may reflect either abnormal TSPO density or a mere shift in possibly normal, relative distribution between the two tissue regions. The most useful pseudoreference region is therefore one in which the true regional density of TSPO is unchanged in the study population, and is yet unidentified in schizophrenia. Building on this biology and methodology, we discuss misconceptions about imaging TSPO in psychosis and cautiously remind the field that this technique should not be equated with ‘imaging microglial activation’ or ‘imaging neuroinflammation.’ Indeed, PET-based TSPO estimates have not correlated with increased peripheral or central pro-inflammatory cytokine levels in schizophrenia or other psychiatric diseases like major depressive disorder.

**Discussion:**

Together, a less simplified approach to imaging TSPO may inform its utility in studying other biological processes captured by its use in psychosis, and may guide future, complimentary research in vitro and in vivo to enhance our understanding of altered neuroimmune processes in psychosis.

